# Neotropical termite microbiomes as sources of novel plant cell wall degrading enzymes

**DOI:** 10.1038/s41598-020-60850-5

**Published:** 2020-03-02

**Authors:** Matias Romero Victorica, Marcelo A. Soria, Ramón Alberto Batista-García, Javier A. Ceja-Navarro, Surendra Vikram, Maximiliano Ortiz, Ornella Ontañon, Silvina Ghio, Liliana Martínez-Ávila, Omar Jasiel Quintero García, Clara Etcheverry, Eleonora Campos, Donald Cowan, Joel Arneodo, Paola M. Talia

**Affiliations:** 1 Instituto de Agrobiotecnología y Biología Molecular (IABIMO), Instituto Nacional de Tecnología Agropecuaria (INTA), Consejo Nacional de investigaciones Científicas y Tecnológicas (CONICET), Hurlingham, Buenos Aires, Argentina; 2 Cátedra de Microbiología Agrícola, Facultad de Agronomía, Universidad de Buenos Aires, INBA-CONICET, Ciudad Autónoma de Buenos Aires, Argentina; 3 Centro de Investigación en Dinámica Celular, Instituto de Investigación en Ciencias Básicas y Aplicadas, Universidad Autónoma del Estado Morelos, Cuernavaca, Morelos, Mexico; 4 Biological Systems and Engineering Division, Lawrence Berkeley National Laboratory, Berkeley, CA USA; 5 Department Biochemistry, Genetics and Microbiology, Centre for Microbial Ecology and Genomics, University of Pretoria, Pretoria, South Africa; 6 Biología de los Invertebrados. Facultad de Ciencias Exactas y Naturales y Agrimensura. Universidad Nacional del Nordeste, Corrientes, Argentina

**Keywords:** Next-generation sequencing, Metagenomics

## Abstract

In this study, we used shotgun metagenomic sequencing to characterise the microbial metabolic potential for lignocellulose transformation in the gut of two colonies of Argentine higher termite species with different feeding habits, *Cortaritermes fulviceps* and *Nasutitermes aquilinus*. Our goal was to assess the microbial community compositions and metabolic capacity, and to identify genes involved in lignocellulose degradation. Individuals from both termite species contained the same five dominant bacterial phyla (Spirochaetes, Firmicutes, Proteobacteria, Fibrobacteres and Bacteroidetes) although with different relative abundances. However, detected functional capacity varied, with *C. fulviceps* (a grass-wood-feeder) gut microbiome samples containing more genes related to amino acid metabolism, whereas *N. aquilinus* (a wood-feeder) gut microbiome samples were enriched in genes involved in carbohydrate metabolism and cellulose degradation. The *C. fulviceps* gut microbiome was enriched specifically in genes coding for debranching- and oligosaccharide-degrading enzymes. These findings suggest an association between the primary food source and the predicted categories of the enzymes present in the gut microbiomes of each species. To further investigate the termite microbiomes as sources of biotechnologically relevant glycosyl hydrolases, a putative GH10 endo-β-1,4-xylanase, Xyl10E, was cloned and expressed in *Escherichia coli*. Functional analysis of the recombinant metagenome-derived enzyme showed high specificity towards beechwood xylan (288.1 IU/mg), with the optimum activity at 50 °C and a pH-activity range from 5 to 10. These characteristics suggest that Xy110E may be a promising candidate for further development in lignocellulose deconstruction applications.

## Introduction

Over the past two decades, the global interest in the development of renewable energies has increased dramatically, particularly in the context of the climate change and the depletion of fossil fuels^1,2^. Bioethanol is considered a valuable renewable energy source capable of providing an alternative to petrol through blending with gasoline^2,3^. The main drawback with the production of bioethanol from lignocellulosic material is the cost of enzymatic hydrolysis because of low catalytic efficiencies of the enzymes currently in use^1,2,4^. A low-cost lignocellulose hydrolysis strategy is also of interest in other processes such as found in the textile, food, animal feed and paper industries^1,5^.

Lignocellulosic biomass mainly consists of polysaccharide polymers, cellulose and hemicellulose, and the phenolic polymer lignin. Termites are among the most efficient lignocellulose decomposers on earth, with hydrolysis efficiencies of up to 90%^6–9^. The ability of termites to degrade lignocellulose is more efficient than the digestion of less lignified forage grasses in ruminants^6,10,11^. This capacity to degrade lignocellulose with very high efficiency is due to a dual system that includes the mechanical and enzymatic machinery of the termite host, together with the action of intestinal symbionts^6,9,12^. This metabolic potential makes termites an ideal target to search for microbial lignocellulosic enzymes that might be used in the textile, food, animal feed, paper and biofuel industries^9,13^.

A wide range of microbial enzymes within the termite gut have been categorized in the different Carbohydrate-Active enZYmes (CAZy) classes (http://www.cazy.org/)^14^, including glycoside hydrolases (GHs), glycosyltransferases (GTs), polysaccharide lyases (PLs), carbohydrate esterases (CEs), carbohydrate-binding modules (CBMs) and auxiliary activity enzymes (AAs)^15–17^. Among these enzymes, some GHs families are particularly important for lignocellulosic biomass deconstruction, most especially in cellulose and hemicellulose degradation. For example, endo-β-1,4-glucanases, exo-β-1,4-glucanases or cellobiohydrolases, and β-glucosidases^18,19^ act in different sections of the cellulose polymer and its derived products, whereas endo-β-1,4-xylanases participate in the degradation of the xylan backbone, the main component of hemicellulose, to xylose. Other GHs families attack different substrates depending on the composition of the polysaccharide or its side chains, including α-L-arabinofuranosidases, endo-α-1,5-arabinanases, endo-β-1,4-mannanases, α-glucuronidases and α-L-fucosidases^20,21^. To date, there have been no reports of termite gut microbiome-derived enzymes that participate in lignin degradation, although this degradation process is known to occur^20,22,23^. The high alkalinity in termite gut segments may act as an alkaline pretreatment to facilitate subsequent lignin degradation^24–26^. It is noted that the anoxic conditions in some regions of the termite digestive tract may not support aerobic lignin degradation pathways^10,27^. It has been proposed that the ligninolytic capacity may be provided by termite host-derived enzymes, although this suggestion remains unresolved^13,20^.

Over the past 15 years, a substantial number of metagenomic, metatranscriptomic and metaproteomic studies of the gut microbiomes of termites and other wood-feeding insects have been reported, with the discovery of numerous enzymes involved in lignocellulose degradation^20,21,28–32^. A number of these genes have been cloned and their enzymatic activities characterized^33–37^. However, until July 2019 only two works referenced in PubMed has reported the cloning and expression of genes encoding GHs from termite gut microbiomes using shotgun metagenome sequencing^15,36^.

In this study, we selected two species of higher termites belonging to the Nasutermitinae subfamily with different life habits and diets (*Cortaritermes fulviceps* and *Nasutitermes aquilinus*). *C. fulviceps* builds mounds and is a polyphagous insect that feeds on leaves, roots and stems of various gramineous plants, as well as wood. By contrast, *N. aquilinus* inhabits live and dead trees and is a strict wood-feeder (monophagous) that consumes hardwoods or softwoods in dry, wet or decaying state^38^.

Our strategy involved an analysis of the gut bacterial diversity of the two termite colonies by using a sequence-driven metagenomic approach. This analysis allowed the identification of candidate genes coding for lignocellulose degrading enzymes. We also performed a comparative analysis of protein structures in a subset of GHs and selected and cloned a GH10 for further biochemical characterisation of enzyme functionality.

## Materials and Methods

### Termite sampling site

Worker caste specimens of the termite species *N. aquilinus* and *C. fulviceps* were collected from single colonies in the province of Corrientes, Argentina (S 27°28′30″: W 58°46′59.43″ and S 27°26′58.26″: W 58°44′17.64″, respectively). *N. aquilinus* and *C. fulviceps* were collected from live *Enterobolium contortisiliquun* trees and from inside a mound located in *Elionurus muticus* grassland, respectively.

The termites were collected with the authorization of the Direction of Natural Resources of Ministry of Tourism of the province of Corrientes (permission number 845/13). No endangered or protected species were used in this study. The specimens were stored at −20 °C until processing. A complete description of the sampling site is reported in Ben Guerrero *et al*.^39^.

### DNA extraction and shotgun sequencing library preparation

The surface of the insects was disinfected with 70% ethanol before dissecting the whole guts under a binocular microscope. Ten guts per termite species were pooled and placed in tubes containing RNA-later (Ambion, Grand Island, USA).

For DNA shotgun sequencing, total genomic DNA was isolated using the DNeasy Blood and Tissue kit (Qiagen, USA) following the manufacturer’s instructions. Crude DNA extracts were further purified according to the QIAmp DNA micro protocol for tissues (Qiagen, USA). The DNA concentration was determined with a Qubit fluorometer (Qiagen) and the size range assessed using a bioanalyzer and gel electrophoresis. The extracted DNA from each sample was diluted to a final concentration of 200 ng for the preparation of a metagenomic library at the QB3 Vincent J. Coates Genomics Sequencing Laboratory (Berkeley, USA). Briefly, DNA was sheared using Covaris and the library preparation was performed using the Kapa Biosystems Library Prep system on the IntegenX Apollo 324 robot. Each sample was sequenced using an Illumina platform (HiSeq 2500 Rapid Run) to generate 150 bp paired-ends reads.

### Metagenomic assembly and annotation

Illumina adapters were removed from the reads and their quality checked using FastQC with the default settings. Paired-end reads were exported to the KBase^40^ (KBase: The U.S. Department of Energy Systems Biology Database) for co-assembly and binning. A random subsample of 20 million paired-end reads per sample was used for assembly using the IDBA-UD assembler (http://i.cs.hku.hk/~alse/h7kubrg/projects/idba_ud/)^41^. Gene abundance was assessed using Bowtie 2 version 2.3.3^42^ to map sequence reads to the assembled contigs and to quantify the number of reads per contig. The coverage was calculated on every contig using gene length estimates, on a coding sequence basis. The coverage information and taxonomy classification were used to investigate the compositions of gut prokaryote communities of the two termite colonies.

Obtained scaffolds were RPKM (Reads per kilobase per Million) normalized to account for sequencing depth and scaffold length. The number of reads for each assembled contig in each sample was normalized to reads per kilobase per million reads mapped. RPKM-normalized coverage values were used as proxies for the abundance of each scaffold/contig in a sample. Open reading frames were predicted from the final set of scaffolds using Prodigal’s^43^ meta procedure (-p meta).

All obtained contigs were assigned a taxonomic classification label using the Kaiju web application (https://github.com/bioinformatics-centre/kaiju)^44^ and coding sequences annotated using Prokka (http://www.vicbioinformatics.com/software.prokka.shtml)^45^.

The obtained contigs with a minimum length of 1,000 base-pairs were binned using MaxBin2^46^ and the generated genome populations (here referred to as bins) were analysed using CheckM^47^ to assess genome quality. Thirty-three bins were obtained and 17 of this bins likely originated from a single bacterial strain/population with completeness ranging from 5–97%. They were further taxonomically phylotyped according to their corresponding predicted proteins using CheckM reference markers. Only these 17 genome bins and their associated glycoside hydrolases (GHs) were used to analyse the differences in GH abundances between the studied termites. Coverage information for the scaffolds of each genome was extracted from the calculated coverage data RPKM normalized for each scaffold in the metagenome. Bins abundances in each gut sample were calculated as the average RPKM-coverage value over all the scaffolds in a bin. Statistical significance of the coverage distribution of the identified genes was assessed using the Kruskal-Wallis test and pairwise comparisons, which were carried out using the Wilcox test and the Benjamini-Hochber method for p-value adjustment using R software.

Additional functional and KEGG (Kyoto Encyclopedia of Genes and Genomes) metabolic pathway annotations were determined with the KAAS web tool (http://www.genome.jp/tools/kaas/)^48^, to identify the high level functions and utilities of the biological systems. Putative genes involved in plant biomass degradation were identified by comparing the predicted ORFs with the protein families classified in the CAZy database using the web server dbCAN (http://csbl.bmb.uga.edu/dbCAN/annotate.php)^49^.

### Data handling and statistical analyses

The R statistical software version 3.4 was used to filter, process and consolidate data obtained from different servers and software and for all statistical analyses.

### 3D modelling of glycosyl hydrolases

A 3D modelling analysis was performed for 26 putative GHs. Phylogenetic analysis of each GH family was done for the selection of protein-coding gene sequences that would be used for structural modelling. CAZy reference sequences were included together with sequences from our dataset that were assigned to the same family according to dbCAN. One sequence per family was chosen based on its dissimilarity to the reference set (phylogenetic trees not shown).

Subsequently, 3D modelling rounds with no restrictions were made using the I-TASSER server (https://zhanglab.ccmb.med.umich.edu/I-TASSER/)^50^. A 3D model was built for each sequence obtained from the gut metagenomic data, and close structural neighbours were identified. The Visual Molecular Dynamic program was used to visualize the 3D models^51^.

### Cloning, expression and enzymatic activities assays

Assessments of the quality of sequence assembly for the identification of lignocellulose-degrading enzymes were performed by selecting a predicted GH-coding gene KBCPBGKF 45352, here termed Xyl10E. The criteria for the selection was as follows: the gene a) was predicted as able to deconstruct hemicellulose, which is one of the main components of plant cell walls, b) encoded a protein from a highly enriched GH family in the metagenomes, c) had a complete coding sequence containing identifiable start and stop codons and a complete open reading frame, d) contained a signal peptide in its gene product, which suggests it may encode a secreted enzyme, e) lacked transmembrane regions.

For PCR amplification and cloning of the Xyl10E gene, DNA was extracted from *N. aquilinus* gut samples using the QIAamp DNA Stool kit (Qiagen) with modifications. Briefly, pooled gut samples from six individuals were heated at 95 °C in 1 mL of kit lysis buffer, and then ground with a FastPrep protocol (3 cycles of 20 sec. at 6000 rpm) using 300 mg of 150–212 µm glass beads (Sigma, USA). After elution of DNA, an additional purification step was performed with Agencourt AMPure XP magnetic beads (Beckman Coulter, USA). For this purpose, 1.5 volumes of bead solution were added per sample, followed by 5 min magnet incubation and two ethanol 80% washes. Finally, the samples were incubated for 5 min in 50 µL Qiagen elution buffer before eluting the supernatants. DNA concentration and purity were assessed with Qubit® fluorometer. The Xyl10E sequence was amplified, without the native signal peptide, using specific primers (designed from assembled contigs) containing *Bam*HI and *Xho*I restriction enzyme sites: Xyl10E-F: 5′ GGATCCTTCTGCGCCTGACA 3′, Xyl10E-R: 5′ CTCGAGCTATTCCACCAATTTCC 3′, for N-terminal fusion to a 6xHis tag (restriction sites are shown underlined). The amplification product was first cloned in pGEM-T Easy vector using *E. coli* DH5-α competent cells. Then, the plasmid inserts from selected colonies were cloned into pET28b(+) vector (*Bam*HI/*Xho*I) and transformed into competent *E. coli* Rossetta cells. Xyl10E protein expression was induced with 0.5 mM IPTG for 16 h at 37 °C. After cell lysis and sonication (six pulses of 10 s, 28% amplitude), recombinant protein was purified in the soluble fraction with Ni-NTA agarose resin (Qiagen), using 50 mM NaH_2_PO_4_, 300 mM NaCl, 250 mM imidazole, pH 8 as elution buffer. Typically, 1.5 mg/mL of recombinant Xyl10E protein was obtained from 90 mL induced *E. coli* culture.

Enzyme activity assays were performed using the enzyme (diluted in appropriate buffer) at a final concentration of 7.5 μg/mL. Endo-β-1,4-xylanase and endo-β-1,4-glucanase activities were determined in triplicate in microtube assays. For this purpose, 50 μL of purified protein were combined with 50 μL of beechwood xylan (Sigma) (1% w/v) or 50 μL of carboxymethyl cellulose (CMC) (Sigma) (2% w/v) in 0.1 M citrate buffer (pH 6) and incubated for 20 min at 50 °C in a Thermomixer (Eppendorf). Reducing sugars released from the polysaccharide hydrolysis were measured by dinitrosalicylic acid (DNS)^52^ with xylose or glucose standard curves. Specific activity was calculated per mg of total protein (IU/mg). For all enzymatic assays, one international unit (IU) was defined as the amount of enzyme that released 1 µmol of product per minute under the specified assay conditions.

## Results

### Assembly and analysis of metagenomic sequencing data

The analysis of the intestinal DNA extracted from *C. fulviceps* and *N. aquilinus* specimens generated 52 Gb of sequence reads. The reads were assembled into 86,012 and 72,572 contigs for *C. fulviceps* and *N. aquilinus*, respectively (Table 1). Eubacteria accounted for 92.7% (*C. fulviceps*) and 98.2% (*N. aquilinus*) of all classified contigs. Other less represented taxonomic groups included Eukaryote (protists (1.6%), fungi (1.7%) and viridiplantae (0.2%)), virus (0.2%) and Archaea (0.5%) on average for both termite microbiomes. DNA insect contamination was low (4%).

**Table 1:** Summary of sequencing and assembling obtained from gut microbiomes of *C. fulviceps* and *N. aquilinus* specimens.

Parameters	*C. fulviceps*	*N. aquilinus*
Total number of reads	20,000,000	20,000,000
Number of contigs	86,012	72,572
Total of contig sequences (Mb)	78.72	105.05
N50 contig reads	26,314	13,545
N90 contig reads	71,366	54,228
Mean contig length	915	1447
Median contig length	726	912
Longest contigs	30,128	119,369
Shortest contigs	400	400

**Figure 1:**
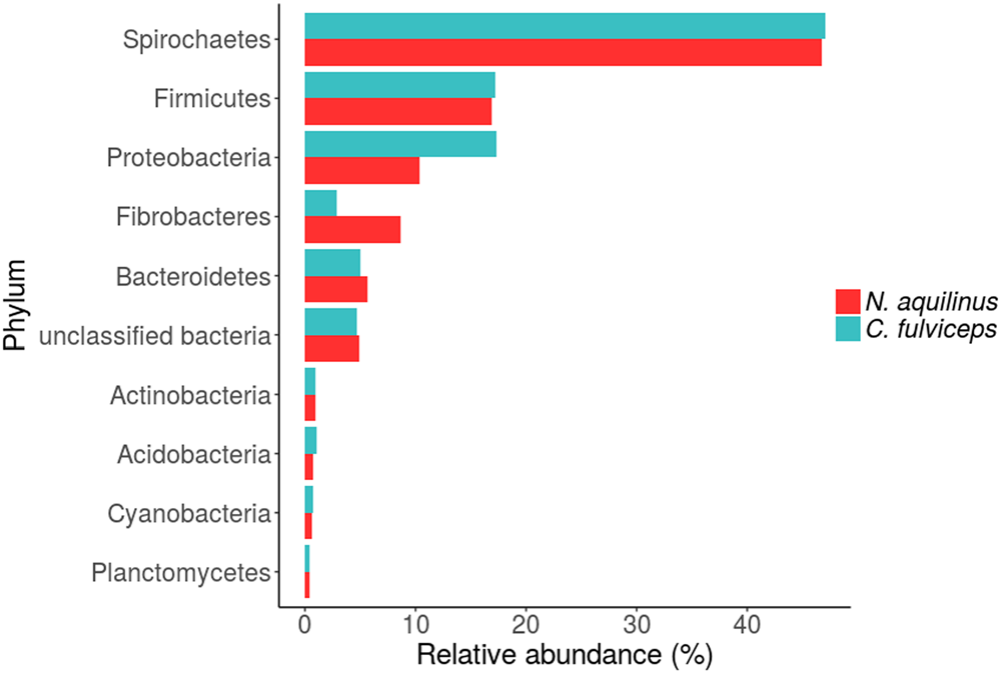
Relative abundance, according to read count, of bacterial phyla in the gut of colonies of *C. fulviceps* and *N. aquilinus*.

We then analysed the microbial community compositions and determined the abundances of bacterial phyla in the guts of both termite colonies. Spirochaetes, Firmicutes, Proteoabacteria, Fibrobacteres, Bacteroidetes and Actinobacteria were the most abundant phyla in both termite gut microbiomes (Fig. 1). Both microbiomes showed a low percentage of unclassified bacteria (~4.8%). The five dominant phyla were the same for both termites’ samples, with only minor differences in the relative abundances. In *C. fulviceps*, the most abundant phylum was Spirochaetes (47%), followed by Proteobacteria and Firmicutes (almost in equal proportions, *ca*. 17%), Bacteroidetes (5%) and Fibrobacteres (2%) and *N. aquilinus* with Spirochaetes (47%), Firmicutes (17%), Proteobacteria (10%), Fibrobacteres (8%) and Bacteroidetes (5%) as the predominant phyla (Fig. 1). Given that the analysis involved only single composite metagenomes from each termite species, no statistical significance can be assigned to these differences. The bacterial phyla, Actinobacteria, Acidobacteria, Cyanobacteria and Planctomycetes, all accounted for more than 2% of the total sequences reads.

Altogether, these nine phyla represented 92.5% and 91.1% of total reads in the gut microbiomes of the *C. fulviceps* and *N. aquilinus* specimens, respectively.

### Comparative functional and metabolic analysis

**Figure 2:**
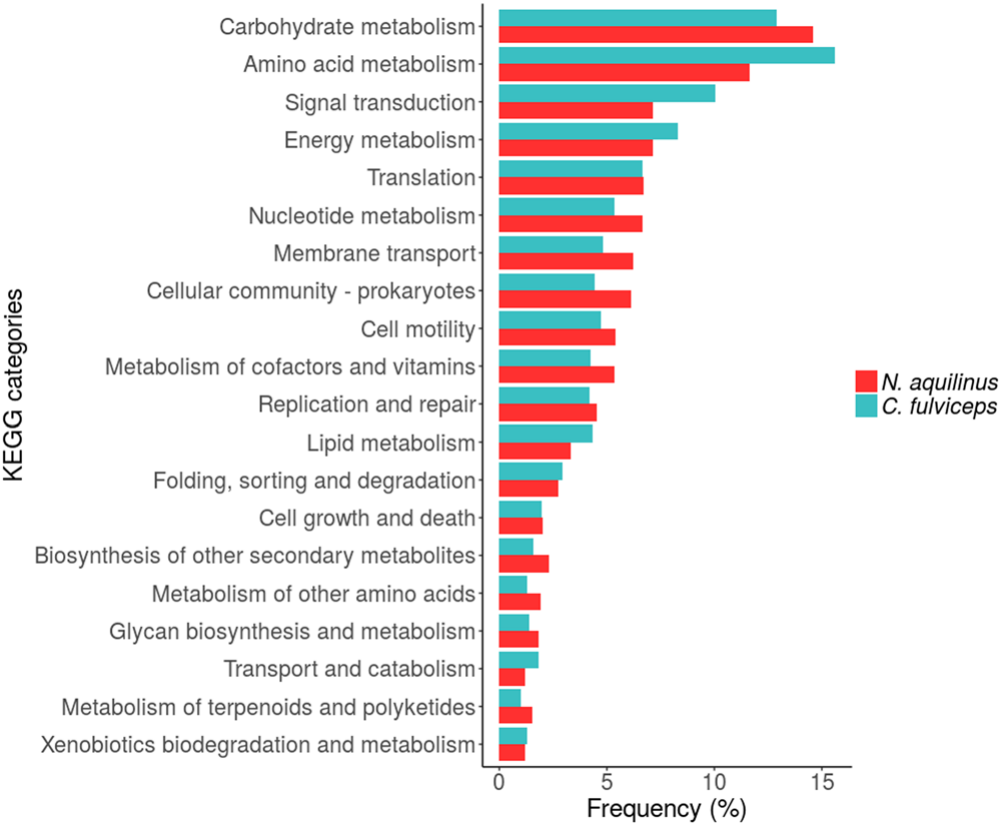
Metabolism pathway classification of the predicted proteins from termite guts samples.

To investigate the diversity of microbial enzymes in the gut samples, we annotated open reading frames (ORFs) in contigs using Prokka and assigned the predicted proteins to metabolic pathways with KEGG. We assessed the metabolic functions of gut microbiomes from the two termite colonies and classified 20 different categories with frequency of more than 2% (Fig. 2).

Annotations relating to amino acid metabolism (AAM) and carbohydrate metabolism (CM) accounted for *ca*. 30% of all predicted ORFs, regardless of the termite sample (Fig. 2). Interestingly, predicted AAM genes were the most abundant in the *C. fulviceps* gut microbiome, whereas ORFs involved in CM dominated in the *N. aquilinus* sample.

### Cellulose and xylan degradation

To further explore the potential capacity of the gut microbiota to degrade lignocellulosic substrates, we screened the putative encoded protein sequences using the Carbohydrate-Active Enzymes (CAZymes) catalogue via the dbCAN annotation web server. We detected 585 and 1,967 putative CAZymes corresponding to 117 and 160 different families, in the *C. fulviceps* and *N. aquilinus* gut microbiome samples, respectively (Table 2). GHs, GTs, CBMs and CEs classes were the most abundant CAZyme categories, together represented 95.9% (*C. fulviceps*) and 97.4% (*N. aquilinus*) of all the CAZyme related annotations. Glycoside hydrolases were the most highly represented enzyme class with 40.3% (*C. fulviceps*) and 37.6% (*N. aquilinus*) of total ORFs. According to the dbCAN database analysis, the 975 GHs corresponded to 71 different CAZy families.

**Table 2:** CAZy classification of predicted ORFs from *C. fulviceps* and *N. aquilinus* gut samples.

CAZy Modules Classification	Total families	*C. fulviceps*			*N. aquilinus*		
		#Families	#ORFs	% ORFs	#Families	#ORFs	% ORFs
Glycoside Hydrolases (GHs)	71	49	236	40.34	65	739	37.57
Glycosiltransferases (GTs)	38	24	140	23.93	32	598	30.40
Carbohydrate Binding Modules (CBMs)	44	29	111	18.97	42	435	22.11
Carbohydrate Esterases (CEs)	14	9	74	12.65	13	145	7.37
Auxiliary Activity (AAs)	3	2	17	2.91	2	28	1.42
Polysaccharide Lyases (PLs)	5	3	6	1.03	4	17	0.86
S-layer homology domain (SLH)	1	—	—	—	1	1	0.05
Dockerin	1	1	1	0.17	1	4	0.20
Total	177	117	585	100	160	1,967	100

**Figure 3:**
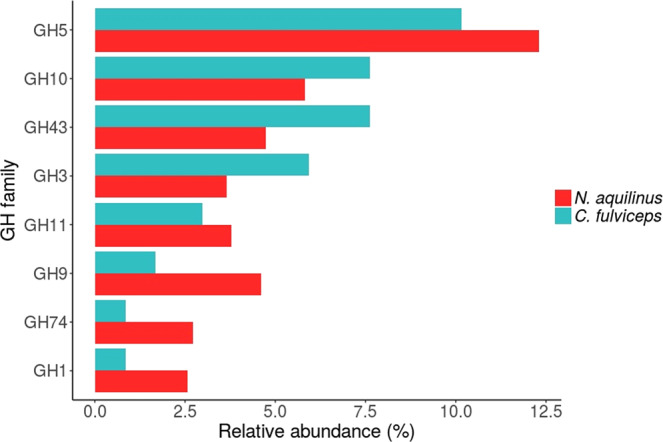
Comparison of the most abundant predicted ORFs in GHs families.

The grouping proposed by Allgaier *et al*.^53^ arranges all known GHs according to their main functional role (cellulases, hemicellulases, debranching enzymes, oligosaccharide degradation, and cell wall elongation). Following this approach, we identified 21 and 26 families that were potentially involved in lignocellulose degradation in the *C. fulviceps* and *N. aquilinus* samples, respectively (Table 3). The frequencies of these different functional groups were similar for the gut microbiome samples of the two termites. For both termite gut samples, the most abundant groups were cellulases and oligosaccharide-degrading enzymes. Three cellulase families (GH5, GH9 and GH45) were present in both termite specimens, whereas GH44 was only found in the *N. aquilinus* gut sample. In both termite colonies, the most abundant ORFs corresponded to GH5 cellulases. Other GHs included in the top eight lignocellulose-degrading enzymes were hemicellulases (GH10 and GH11), oligosaccharide-degrading enzymes (GH43, GH3 and GH1), cellulases (GH9) and cell wall elongation enzymes (GH74) (Fig. 3).

**Table 3:** Inventory of glycoside hydrolases (GHs) related to lignocellulose degradation in the gut of *C. fulviceps* and *N. aquilinus* samples.

CAZy Family	Main known activities (CAZy)	*C. fulviceps* (%)	*N. aquilinus* %	ID number	Pfam ID
**Glycoside Hydrolase catalytic domain**					
**Cellulases**					
GH5	Cellulase, β-1,4-endoglucanase, β-1,3-glucosidase, β-1,4-endoxylanase, β-1,4-endomannanase, exo-β-1,4-glucanase, others	10.1	12.1	KBCPBGKF 16418	PF00150
GH9	endoglucanase, cellobiohydrolase, β-glucosidase	1.7	5.2	KBCPBGKF 25469	PF00759
GH44	endoglucanase, xyloglucanase	0	0.3	KBCPBGKF 27594	PF12891
GH45	endoglucanase	1.7	1.5	KBCPBGKF 22078	PF02015
Subtotal (%)		13.5	19.1		
**Hemicellulases**					
GH8	cellulase, endo-1,4-β-xylanase	0.4	1.1	KBCPBGKF 08463	PF01270
GH10	β-1,4-xylanase, β-1,3-xylanase	7.6	5.7	KBCPBGKF 45352	PF00331
GH11	β-1,4-xylanase, β-1,3-xylanase	3	3.7	KBCPBGKF 39042	PF00457
GH26	β-1,3-xylanase, mannanase	0.8	1.5	KBCPBGKF 06103	PF02156
GH28	polygalacturonase, rhamnogalacturonase, others	0.4	0.1	KBCPBGKF 36314	PF00295
GH53	β-1,4-endogalactanase	0.8	0.8	AFHCADON 03545	PF07745
Subtotal (%)		13	12.9		
**Debranching enzymes**					
GH51	α-L-arabinofuranosidase, endoglucanase, β-xylosidase, endo-β-1,4-xylanase	0.8	0.7	KBCPBGKF 11251	PF06964
GH67	α-glucuronidase, xylan α-1,2-glucuronidase	1.3	0.4	AFHCADON 05433	PF07488
GH78	α-L-rhamnosidase, rhamnogalacturonan α-L-rhamnohydrolase	0.4	0.1	KBCPBGKF 28234	PF17389
GH106	α-L-rhamnosidase	0	0.8	KBCPBGKF 10146	PF17132
GH115	xylan α-1,2-glucuronidase	0	0.4	KBCPBGKF 12414	PF15979
Subtotal (%)		2.5	2.4		
**Oligosaccharide-degrading enzymes**					
GH1	β-glucosidase, β-galactosidase, β-mannosidase, others	0.8	2.5	KBCPBGKF 11626	PF00232
GH2	β-galactosidase, β-mannosidase, others	0.4	0.5	AFHCADON 00817	PF02836
GH3	β-1,4-glucosidase, β-1,4-xylosidase, β-1,3glucosidase, α-L-arabinofuranosidase, others	5.9	3.6	AFHCADON 00942	PF00933
GH29	α-L-fucosidase	0.4	0.4	AFHCADON 20026	PF01120
GH38	α-mannosidase	0	0.1	KBCPBGKF 23895	PF01074 PF07748
GH39	α-L-iduronidase, β-xylosidase	1.7	1.1	KBCPBGKF 02663	PF00150
GH42	β-galactosidase, α-L-arabinopyranosidase	1.3	0.9	AFHCADON 00278	PF02449
GH43	xylanase, β-xylosidase, α-L arabinofuranosidase, arabinanase, others	7.6	4.7	KBCPBGKF 09493	PF04616
GH52	β-xylosidase	0	0.5	KBCPBGKF 07354	PF03512
Subtotal (%)		18.1	14.3		
**Cell Wall elongation 16 17, 74, 81**					
GH16	β-1,3(4)-endoglucanase, other	0.8	0.8	KBCPBGKF 11096	PF00722
GH74	endoglucanase, cellobiohydrolase, xyloglucanase	0.8	3.9	KBCPBGKF 51788	PF00754
Subtotal (%)		1.6	4.7		

GHs are arranged according to the major functional role as grouped by Allgaier *et al*. (2010). Relative abundance (%) of de GHs families were included in the analysis.

An association between a higher relative abundance of Spirochaetes and an increased level of cellulose degrading enzymes has been previously reported. To test this, we extracted genomes from the metagenomes of the analysed termites based on coverage and composition. A total of 33 genome populations were binned, with 17 of these genomes showing a completeness of 5–97% and identified as consisting only of bacterial elements. Glycoside hydrolases and their corresponding normalized coverage were extracted from the binned genomes and differences in their abundances between the two termite samples analysed (Fig. S1). The obtained results showed a significantly higher abundance of genome-associated GHs in *N. aquilinus* than in *C. fulviceps*. This finding was further evaluated by comparing the normalized average-coverage of the extracted genomes (Fig. S2).

### 3D modelling analysis of glycosyl hydrolases

Twenty six putative GH sequences obtained from the metagenomic analysis were modelled using I-TASSER (Table 4). Each modelled sequence was associated to a GH family based on both the templates and structural analogues identified, as deposited in the PDB database. All 26 sequences were identified as GHs and grouped in 23 different families, according to their structural properties. For most, the identification was in accordance with the CAZy classification reported in Table 3. Nevertheless, the predicted proteins AFHCADON 03545, KBCPBGKF 10146 and KBCPBGKF 23895, which were identified as GH 53, 106 and 38 (CAZy classification), respectively, were identified as members of GH1 and GH26 families according to protein modelling.

**Table 4:** Summary of data of selected protein structural models.

Reference	C-score	TM	RMSD (Å)	Family asociated	Templates (PDB:ID)	Structural analogs (PDB:ID)	Molecular Weight (kDa)*
KBCPBGKF_11626	1.08	0.86 ± 0.07	4.8 ± 3.1	GH1	5GNX, 1OIF, 5IDI, 2DGA	1OIF, 5IDI, 3TA9, 5GNX	51.08
KBCPBGKF_39042	−2.88	0.39 ± 0.13	14.6 ± 3.7	GH11	1H4G, 2DCJ, 2DCK	2DCJ, 5U70, 1IGO, 2Q1F	57.61
KBCPBGKF_12414	1.79	0.97 ± 0.05	4.9 ± 3.2	GH115	4ZMH, 4C90, 4C91	4ZMH, 4C90, 2VCA, 1GQI	108.5
KBCPBGKF_11096	−0.65	0.63 ± 0.14	8.2 ± 4.5	GH16	3ILN, 1UPS, 2VY0, 3AZY	3ILN, 2VY0, 1UPS, 4DFS	45.6
AFHCADON_00817	1.52	0.93 ± 0.06	5.6 ± 3.5	GH2N	3CZJ, 1BG1, 3DEC, 3BGA	3DYP, 3BGA, 3DEC, 3OB8	116.75
KBCPBGKF_06103	−0.15	0.69 ± 0.12	8.0 ± 4.4	GH26	3ZM8, 2BVY, 3TP4, 2BVT	3ZM8, 2X2Y, 2WHM, 4YN5	67.14
KBCPBGKF_36314	0.26	0.75 ± 0.10	6.5 ± 3.9	GH28	3JUR, 2UVE	3JUR, 2UVF, 1BHE, 4C2L	48.56
AFHCADON_00942	1.16	0.84 ± 0.08	6.0 ± 3.7	GH3C	5A7M, 4ZO6, 5TF0, 3U48	5A7M, 3U48, 4ZO6, 5JP0	76.25
KBCPBGKF_02663	0.55	0.79 ± 0.09	5.7 ± 3.6	GH39	5BX9, 5BXA, 4M29	5BX9, 5JVK, 1UHV, 2BFG	50.7
AFHCADON_00278	2	0.99 ± 0.04	3.6 ± 2.5	GH42	3TTS, 3TTY	3TTS, 5E9A, 4OIF, 4UNI	76.61
KBCPBGKF_09493	0.97	0.85 ± 0.08	5.4 ± 3.4	GH43	5JOW, 1YIF, 5JOW, 1YRZ	1Y7B, 1YIF, 3C2U, 2EXK	60.54
KBCPBGKF_27594	−0.36	0.67 ± 0.13	8.3 ± 4.5	GH44	2YIH, 3IK2, 2YIH, 2E4T	3IK2, 2YJQ, 2EJ1, 3II1	59.29
KBCPBGKF_22078	−1.35	0.54 ± 0.15	10.8 ± 4.6	GH45	3ENG, 4M00, 3ENG, 3WNK	4M00, 2UVC, 1G8X, 5CSK	56.79
KBCPBGKF_16418	−0.82	0.61 ± 0.14	9.1 ± 4.6	GH5	4X0V, 3ICG, 4X0V, 3ICG	3ICG, 4X0V, 1EDG, 3AYR	54.21
KBCPBGKF_11251	1.91	0.99 ± 0.04	3.4 ± 2.4	GH51	1QW9, 2C8N, 1QW9, 1QW9	1QW9, 2C8N, 2Y2W, 3S2C	56.11
KBCPBGKF_07354	1.59	0.94 ± 0.05	4.7 ± 3.1	GH52	4C1O, 4C1P	4C1O, 5FJS, 2CQS, 1V7W	79.82
AFHCADON_03545	−1.17	0.57 ± 0.15	10.5 ± 4.6	GH1	2GFT, 4V2X, 1R8L, 4QAW	1UR4, 4V2X, 5E0C, 4YZP	69.79
AFHCADON_05433	2	0.99 ± 0.03	3.1 ± 2.2	GH67M	1L8N, 1K9D, 1MQQ, 1GQI	1MQQ, 1GQI, 1GQJ, 4C90	76.92
KBCPBGKF_25790	−0.67	0.63 ± 0.14	7.9 ± 4.4	GH74	2XBG, 4LGN, 5FKQ, 5OJ5	2XBG, 5OJ3, 3OKY, 3AL9	36.11
KBCPBGKF_28234	−1.5	0.53 ± 0.15	11.1 ± 4.6	GH78	6GSZ	6GSZ, 3W5M, 6I60, 2OKX	61.51
KBCPBGKF_08463	−2.28	0.45 ± 0.14	13.7 ± 4.0	GH8	5X3A, 5XD0, 1V5D, 1KWF	5X3A, 1V5D, 1CEM, 1H14	75.93
KBCPBGKF_25469	1.85	0.98 ± 0.05	3.6 ± 2.5	GH9	3X17, 1UT9	3X17, 3RX5, 1UT9, 1CLC	57.47
AFHCADON_20026	1.01	0.85 ± 0.08	4.7 ± 3.1	GH29	6GN6, 2ZX9, 4J27, 2WVV	6GN6, 2WVT, 4NI3, 2ZX9	45.22
KBCPBGKF_10146	1.01	0.85 ± 0.08	4.2 ± 2.8	GH26	3ZM8, 3WDQ, 6HPF	3ZM8, 3WDQ, 6HPF, 6HF2	36.96
KBCPBGKF_23895	1.16	0.87 ± 0.07	3.9 ± 2.7	GH26	6HPF, 3ZM8, 3WDQ	3ZM8, 6HPF, 3WDQ, 6HF2	36.74
KBCPBGKF_45352	0	0.71 ± 0.11	6.8 ± 4.1	GH10	6FHF	6FHF, 1HIZ, 2FGL, 2UWF	45.63

In general, the 3D models showed C-score values between −2.88 to 2.00 and TM values from 0.39 to 0.99. The C-score is estimated based on the significance of threading template alignments and the parameters obtained from the structure assembly simulations. The C-score values range from −5 to 2 where higher values indicate a higher confidence model. TM, which varies from 0 to 1, is calculated from the C-score, where the optimum TM value for a 3D model is 1. According to these parameters, the 3D models were generally estimated to be of high quality. However, some 3D models (i.e. models obtained from sequences: KCPBGKF 24894, KBCPBGKF 39042, KBCPBGKF 08463) showed low quality scores according to the C-score and TM values.

As previously noted, GH families 3, 5, 10, and 43 were the most abundant in both termite gut metagenomes. Members of GH family 10 have been extensively studied in terms of their capacity for hemicellulose deconstruction^54–64^. Accordingly, from the 26 modelled GHs, we selected the sequence KBCPBGKF 45352, a GH family 10 enzyme henceforth termed Xyl10E, for further structural analysis and biochemical characterization. The sequence presented 45% identity with a endo-β-1,4-xylanase from *Treponema azotonutricium* (GenBank: AEF82584.1)^65^.

The first attempt to model Xyl10E yielded five possible models (best model: C-score = −0.17; TM = 0.69 ± 0.12 and RMSD = 7.2 ± 4.2 Å). PDBs 2FGL, 6FHF, 4W8L, 2Q8X, 5OFJ and 2UWF (where the code identifies a crystallographic structure deposited in the Protein Data Bank) were the templates used by i-ITASSER to produce Xyl10E 3D-models. Proteins identified as both templates and structural analogues belonged to GH family 10^66^. To obtain a more accurate 3D-model, we performed a second round of modelling and obtained six models by using each identified structural analogue as a separate template. PDB 6FHF was found to be the best template to produce an optimum Xyl10E 3D-model. However, the quality values (C-score = 0; TM = 0.71 ± 0.11; RMSD = 6.8 ± 4.1 Å) obtained during this second modelling round suggested some important structural differences with those PDB identified as structural neighbours. Curiously, 6FHF is an unusual GH10 sequence, because it was designed by using rational protein design approaches, and generated by automated combinatorial backbone assembly and sequence design^67^. This observation supports the importance of using metagenomic approaches for the discovery of enzymes with unusual sequences.

**Figure 4:**
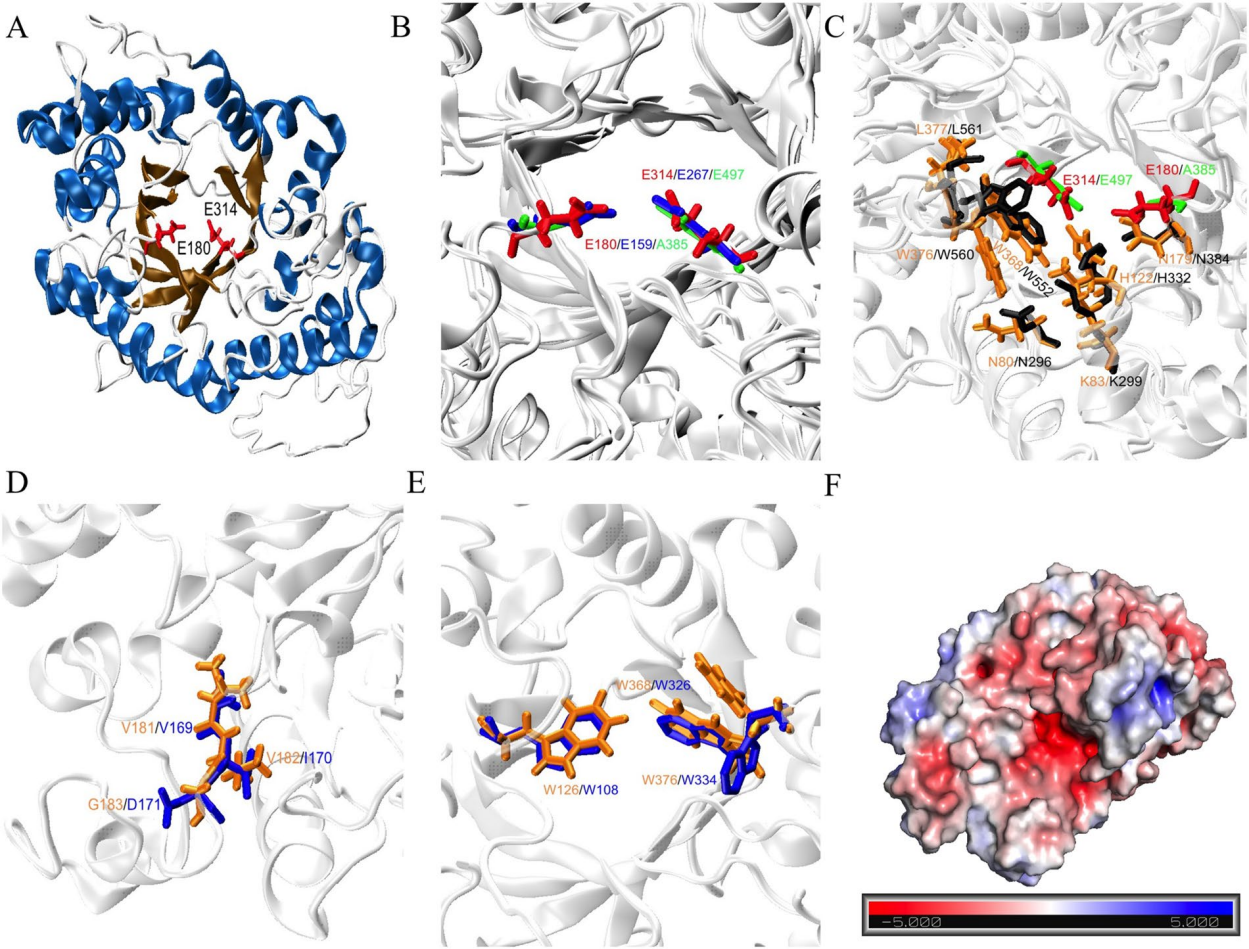
The overall TIM-barrel structure of Xyl10E. (**A**) Catalytic residues are shown in red (E180 and E314). (**B**) Catalytic residues of GH10 family are shown in red, blue and green in Xyl10E, PDB 6FHF, PDB 2W5F, respectively. (**C**) Another seven residues strictly conserved in GH10 are shown in orange and black in Xyl10E and PDB 2W5F, respectively. (**D**) Residues involved in the alkaline sensitive xylanases are shown in orange and blue in Xyl10E and PDB 2UWF, respectively. (**E**) Aromatic residues forming the aromatic cage that surround the catalytic pocket. W108, W326 and W334 (in blue) and W126, W368 and W376 (in orange) belong to PDB 2UWF and Xyl10E, respectively. (**F**) Electrostatic potential of Xyl10E. Negatively and positively charged surfaces are coloured in red and blue, respectively.

The structural analysis of the Xyl10E model (Fig. 4) confirmed the characteristic folding topology of the GH10 family, an eight-fold TIM-barrel structure. Xyl10E showed the catalytic residues, E180 and E314, that are conserved in this family (Fig. 4A,B), as well as the seven conserved residues of the active site (Fig. 4C). Furthermore, this enzyme had one (V169) of three residues involved in the sensitivity to alkaline pH described in the active alkaline xylanase of *Bacillus halodurans* S7 PDB 2UWF^68^ (Fig. 4D) and an aromatic box, which is also characteristic of the GH10 family (Fig. 4E). We also assessed the electrostatic potential of Xyl10E (Fig. 4F) and found that the negative charge is concentrated in the centre of the sequence, which is consistent with the active alkaline xylanase of PDB 2UWF.

### Verification of sequence assembly and evaluation of enzymatic activities

We subsequently validated the predicted data by evaluating the activity of the recombinant Xyl10E. Xyl10E was successfully cloned and expressed in *E. coli* as an N-terminal His-tag fusion protein, and purified in a soluble form that allowed the subsequent functional characterization.

**Figure 5:**
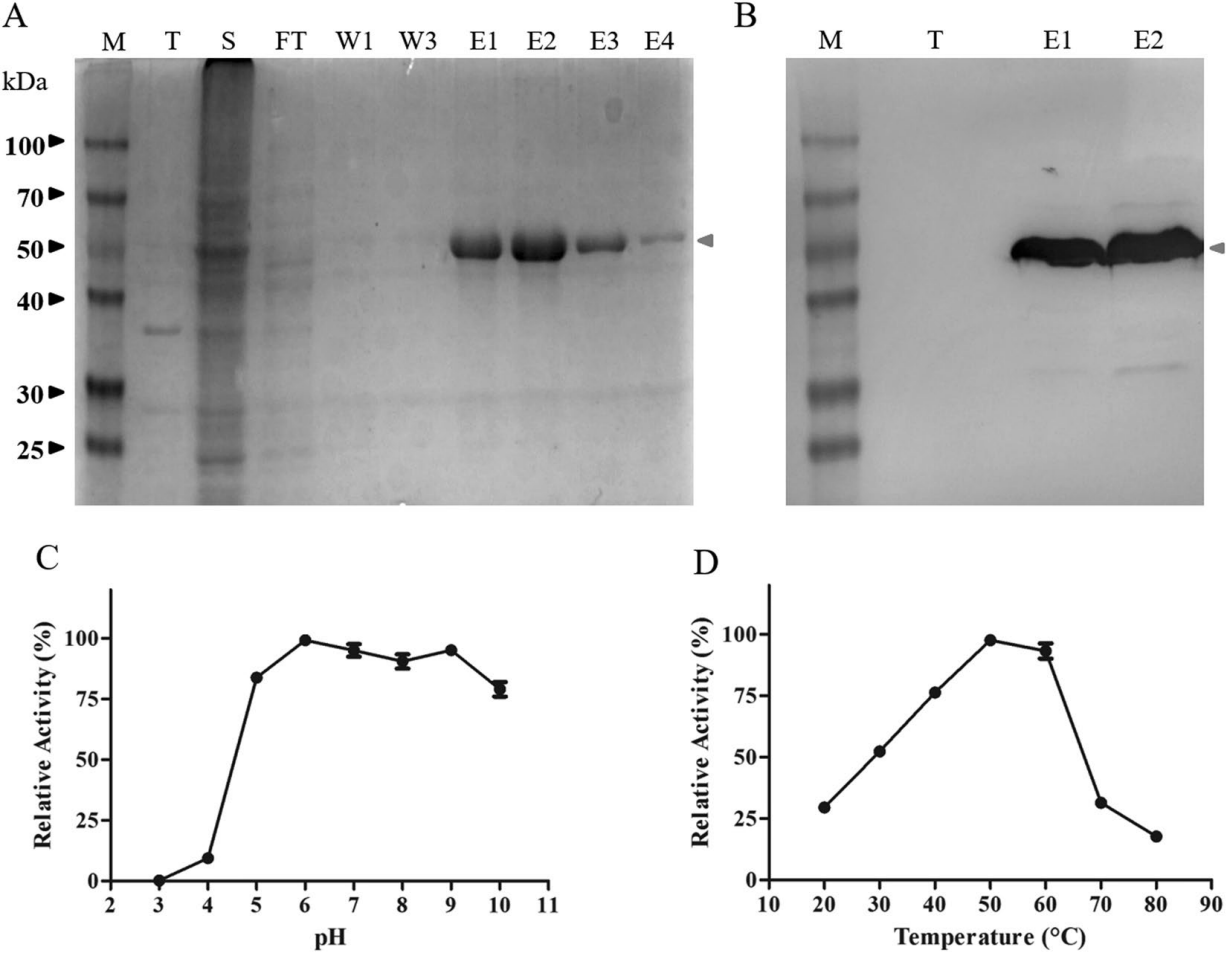
Expression, purification and enzymatic characterization of soluble Xyl10E. (**A**) SDS-PAGE, M: molecular weight marker, T: total protein content of cell lysates without induction, S: soluble fraction of cell lysates, FT: flow through, W1 and W3: washed fractions with 20 mM imidazole, E1 to E4: serial elution fractions with 250 mM imidazole. The arrow indicates the band corresponding to Xyl10E (49.2 kDa). (**B**) Western blot revealed with anti-His antibody and peroxidase activity. (**C**,**D**) Effect of pH and temperature on the recombinant xylanase.

The predicted molecular weight (MW) and isoelectric point (IP) of recombinant Xyl10E were 49.2 kDa and 6.31, respectively. The purified protein showed an apparent monomeric molecular weight of 49 kDa, in accordance with the predicted size (Fig. 5A,B).

According to the enzymatic activity assays, Xyl10E had a specific endo-β-1,4-xylanase activity of 288.1 IU/mg of enzyme, with an optimum activity at around 50 °C and pH 6. Interestingly, this enzyme retained more than 50% of its optimum activity over a wide temperature range (30 to 60 °C) and more than 80% over a wide pH range (5 to 10) (Fig. 5C,D). Endo-β-1,4-glucanase activity was negligible.

## Discussion

In recent years, the search for new enzymes that degrade lignocellulose has become essential. These enzymes are important for biofuel production and other industries, such as paper, food and textile. In this context, the ability of termites to feed on wood and other types of plant biomass makes them an ideal system to obtain efficient cell wall degrading enzymes^6,9,69,70^. In higher termites, many of the main cellulolytic enzymes are produced by their bacterial endosymbionts. For this reason, a comprehensive exploration of gut microbiota is essential to understand the processes involved in lignocellulose digestion. Here, we characterized the bacterial community hosted in the guts of *C. fulviceps and N. aquilinus* colonies and identified the relevant putative proteins involved in lignocellulose degradation. Spirochaetes, Firmicutes, Proteobacteria, Fibrobacteres and Bacteroidetes were the dominant bacterial phyla in both termite specimens and Spirochaetes accounted for almost half of the sequences present in both termite samples. The same dominant bacterial phyla have been observed in other wood and grass feeders termites such as *Nasutitermes corniger, N. ephratae, Microcerotermes* sp*., N. takasagoensi* and *Mironasutitermes shangchengensis*^20,21,71–74^.

Spirochaetes seems to be important for the survival of higher termites^75,76^, as this group is known to be involved in all of the major functions in the termite hindgut (fibre hydrolysis, fermentation, homoacetogenesis and nitrogen fixation)^20,21,74,77–79^. The dominance of Spirochaetes in the hindgut environment may be linked to their high mobility in viscous media and to the high surface to volume ratio of their cells^6,77^. Studies of the hindguts microbiota of *Nasutitermes* and *Amitermes* spp.^20,21^ have attributed the abundance of glycoside hydrolases putatively involved in cellulose degradation to Spirochaetes. Our results demonstrate that *N. aquilinus* harbours Spirochaetes in higher abundance than *C. fulviceps* (Fig. S2A), and that these *Treponema* sp. genome-bins contain a larger array of putative GHs with functions related to cellulose and hemicellulose degradation (Fig. S2B). Interestingly, some non-homoacetogenic *Treponema* spp. isolated from lower termites degrade cellobiose^80,81^. This finding indicates that they also have an important role in fibre digestion.

In our study, most of the sequences assigned to the phylum Spirochaetes belonged to the genus *Treponema* (71.6% in *N. aquilinus* colony and 68% in *C. fulviceps*). This genus includes protist ectosymbionts and free-living bacteria in the lumen of the hindgut, both participating in the process of reductive acetogenesis to produce acetate, the main nutrient for the termite host^6,82,83^.

*N. aquilinus* feeds exclusively on wood, whereas *C. fulviceps* consumes more nitrogen rich organic matter, including either living or decaying plant tissues^84,85^. This difference in diet may explain the metabolic pathway profiles of each termite colony. Whereas the amino acid metabolism (AAM) was the dominant metabolic function in the *C. fulviceps* gut microbiome, carbohydrate metabolism (CM) was the predominant pathway in *N. aquilinus* gut microbiome, according to the analysis of ORFs assigned after contig annotation.

Other relevant metabolic categories were broadly similar in both termite microbiomes, supporting the hypothesis that the termites gut microbiomes retain a stable core set of metabolic functions.

The CAZy database classifies cellulases and other plant cell wall polysaccharides degrading enzymes into GHs families. Previous reports show that roughly 34% of GHs families contain enzymes that contribute to plant cell wall deconstruction^86^. In this study, we identified 975 GHs, corresponding to 71 different CAZy families.

We subsequently sorted the GHs involved in lignocellulose degradation according to the arrangement proposed by Allgaier *et al*.^53^. This analysis revealed 21 and 26 distinct GHs families present in the *C. fulviceps* and *N. aquilinus* gut microbiomes, respectively. These results are consistent with those reported by He *et al*.^20^, where a study of the gut microbiomes of *Amitermes wheeleri* (dung feeder) and *N. corniger* (wood feeder) identified around 25 GHs families.

The GHs family classifications are based on protein sequence and structure and therefore does not necessarily accurately predict enzyme activities. Most GHs families comprise proteins with different enzymatic activities and proteins with similar activity can be found in different GHs families^87^. Endoglucanases and other GHs involved in cellulose degradation can be found in several GHs families. For example, β-glucosidases are found in six GHs families and cellobiohydrolases are distributed across three GHs families. In consequence, the prediction of CAZyme enzymatic activity based on their sequences alone is difficult^88^.

We identified multiple cellulases, especially from families GH5 and GH9, in the gut sample from *N. aquilinus*. This termite species has a largely wood-based diet, and the high abundance of cellulases in its gut microbiome is consistent with its dietary preference. *C. fulviceps* was collected from inside a mound located in grassland and its gut microbiome was enrich in debranching- and oligosaccharide-degrading enzymes, in particular α-L-arabinofuranosidases (GH3, GH42 and GH43). In general, grass is composed of cellulose fibres surrounded by hemicellulose, mainly xylans, annotated with arabinose residues in the form of arabinoxylans and glucuronoarabinoxylans^89,90^. The high abundance of these GHs families in the *C. fulviceps* gut microbiome is consistent with its feeding habits, which include grass foraging.

The analysis of cellulolytic GHs distribution suggested that the set of enzymes was different in the termite microbiomes. This could be determined by host factors, diet or taxonomic composition. Thus, further research on microbial enzymes from Nasutitermitinae is necessary to better understand how these variables influences the cellulolytic enzyme diversity.

We also investigated the most abundant annotated ORFs belonging to GHs families in the two metagenome sequence datasets. In both termite gut metagenomes, the most abundant cellulolytic ORFs belonged to family GH5. This is a large multigene family that includes endoglucanases (cellulases) and endo-mannanases, as well as exo-glucanases, exo-mannanases, β-glucosidases and β-mannosidases^91^. Our results are comparable with previous reports in *Amitermes wheeleri* and *N. corniger* termites, where GH5 was identified among the most abundant GHs families^20^. Furthermore, a high proportion of hemicellulases identified in the gut microbiomes of both termites belonged to the GH10 family, followed by members of GH11.

The 3D modelling of the 26 selected protein sequences yielded high quality structural models in most cases, with only three proteins yielding low quality 3D models. The sequences of proteins that were identified and 3D modelled revealed similarity with proteins with a wide range of enzymatic activities, including glucosidases, xylanases, cellulases, rhamnogalactosidases, mannanases, xylosidases, laminarinases and arabinofuranosidases. This result demonstrates that the GH sequences identified in these termites represent a valuable resource for the identification of new genes and gene products for possible use in lignocellulose deconstruction.

A structural analysis of the sequence KBCPBGKF 45352 (Xyl10E) revealed that this protein belongs to GH family 10, showing the correct folding topology, the key catalytic residues and the conserved active site residues typical of proteins in the GH10 family. This analysis, in conjunction with the activity data, confirmed conclusively that Xyl10E is an endo-β-1,4-xylanase of the GH10 family capable of functioning at an alkaline pH.

The analysis of the enzymatic activity of xylanase Xyl10E showed that the enzyme had a specific activity of 288 IU/mg. This value is 3-and 22-fold higher than the experimental values for endo-xylanases from *Paenibacillus* sp., rGH10XynA (~100 IU/mg) and HC1 (~13 IU/mg), respectively^55,63^ and 5- and 15- fold higher than that of *Cohnella laevirobosi* HY-21 (~58 IU/mg)^58^ and *Massilia* sp. XynRBM26 (~20 IU/mg)^64^, respectively. Conversely, the specific activity of Xyl10E was of the same order as several xylanases recovered from functional metagenomics analyses, Xyn10N18 derived from bovine rumen (~242 IU/mg)^56^ and SCXyl extracted from sugarcane soil bacteria (~200 IU/mg)^54^ and Xyl-ORF19, from the gut microbiome of termite *Globitermes brachycerastes* exhibited a specific activity of (~114 IU/mg)^57^.

Most of the reported GH10 family xylanases have been recovered from isolated bacteria or from functional screening of metagenomic libraries^56–64,92–95^. This study shows the value of metagenome DNA assemblages as a source of novel enzymes.

The optimal temperature of Xyl10E was 50 °C and this enzyme retained more than 90% of its optimum activity at 60 °C. These temperatures are in the same range as those found in other metagenome-derived xylanases of the GH10 family^54^. Kim *et al*.^60–62^ characterised several endo-xylanases from GH10 family, all of which were cloned from insect endosymbiont bacteria, and showed that their optimal temperatures range from 50 to 70 °C.

The endo-β-1,4-xylanase Xyl10E showed an optimum activity at pH 6 and retained around 80% of its activity between pH 5 and pH 10. This is consistent with the broad pH-activity ranges reported in xylanases of bacterial origin^59,61,64,95,96^.

Many GH10 xylanases showed both endo-β-1,4-xylanase and endo-β-1,4-glucanase activities^93,94,97^, although some enzymes of this family exhibit only xylanase activity: e.g. Xyn10N18 from a bovine rumen metagenomic library^56^ and XynA_MG1_ from chicken cecum^92^. The endoglucanase activity of Xyl10E against CMC was negligible.

This study has demonstrated that the gut microbiomes of these neotropical higher termite species encode a high diversity of enzymes that are potentially involved in plant cell wall degradation. Further study of these genes and their products might reasonably be expected to produce novel sequences, novel enzyme activities and even novel specificities.

## Supplementary information

Supplementary material.

## Data Availability

All sequence data were deposited in the NCBI Sequence Read Archive under the BioProject accession number PRJNA475948.
