# Supplementary material for: Neotropical termite microbiomes as sources of novel plant cell wall degrading enzymes
Source: Sci Rep. 2020 Mar 2;10:3864. doi: 10.1038/s41598-020-60850-5 (PMC7052144; doi:10.1038/s41598-020-60850-5)

Neotropical termite microbiomes as sources of novel plant cell wall degrading enzymes

Matias Romero Victorica[1±], Marcelo A. Soria[2±], Ramón Alberto Batista-García[3], Javier A. Ceja-Navarro[4], Surendra Vikram[5], Maximiliano Ortiz[5], Ornella Ontañon[1], Silvina Ghio[1], Liliana Martínez-Ávila[3], Omar Jasiel Quintero García[3], Clara Etcheverry[6], Eleonora Campos[1], Don Cowan[5], Joel Arneodo[1] and Paola M. Talia[1]*

1 - Instituto de Agrobiotecnología y Biología Molecular (IABIMO), Instituto Nacional de Tecnología Agropecuaria (INTA), Consejo Nacional de investigaciones Científicas y Tecnológicas (CONICET).

2 - Cátedra de Microbiología Agrícola, Facultad de Agronomía, Universidad de Buenos Aires, INBA-CONICET. Ciudad Autónoma de Buenos Aires, Argentina.

3 - Centro de Investigación en Dinámica Celular, Instituto de Investigación en Ciencias Básicas y Aplicadas, Universidad Autónoma del Estado Morelos, Cuernavaca, Morelos, Mexico.

4- Biological Systems and Engineering Division, Lawrence Berkeley National Laboratory, Berkeley, CA, USA.

5- Department Biochemistry, Genetics and Microbiology, Centre for Microbial Ecology and Genomics, University of Pretoria, Pretoria, South Africa.

6 - Biología de los Invertebrados. Facultad de Ciencias Exactas y Naturales y Agrimensura. Universidad Nacional del Nordeste, Corrientes, Argentina.

* talia.paola@inta.gob.ar/ taliapaolam@gmail.com

± Matias Romero Victorica and Marcelo A. Soria contributed equally to the manuscript

Fig. S1. Distribution of the genetic potential for cellulose and hemicellulose degradation in *C. fulviceps* and *N. aquilinus*. Identified glycoside hydrolases were detected in the metagenome of the two termite samples showing a higher coverage of glycoside hydrolases in *N. aquilinus* than in *C. fulviceps*. In each boxplot, a point represents a single gene per category and its detected coverage; the diamond symbols represent the mean. The box boundaries represent the first and third quartiles of the distribution and the horizontal line inside each box represents the median. Boxplots whiskers span 1.5 times the interquartile range of the distribution and large points outside of the whiskers denote outliers. Statistical differences were evaluated with Kruskal-Wallis test and pairwise comparisons were done using a two-sided Wilcox test with P-values adjusted according the Benjamini-Hochberg method.

Fig. S2. (A) Distribution of metagenome extracted genomes from each termite sample. Yellow to red colour gradient indicates increased average coverage of each genome in each analysed termite. (B) Normalized gene content (putative GHs) per genome. Genomes were phylogenetically assigned using microbial single copy protein coding marker genes, and their metabolic potential defined by protein prediction and annotation against the dbCAN databases. Number of detected genes per genome for each of the analysed categories was normalized by genome size to account for differences in genome recovery. White to dark blue colour gradient indicates increased gene number counts in each genome.

Supplementary figure S1

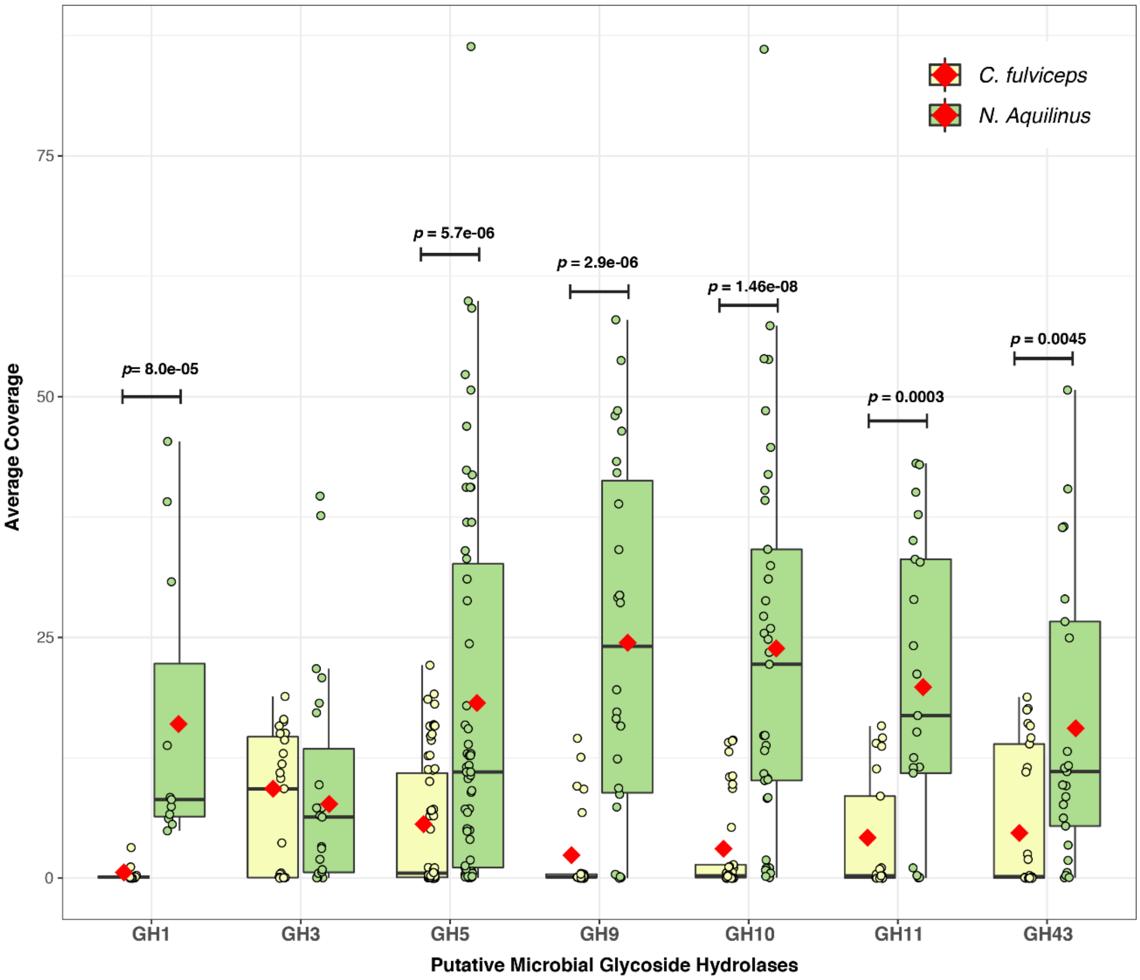

Supplementary figure S2

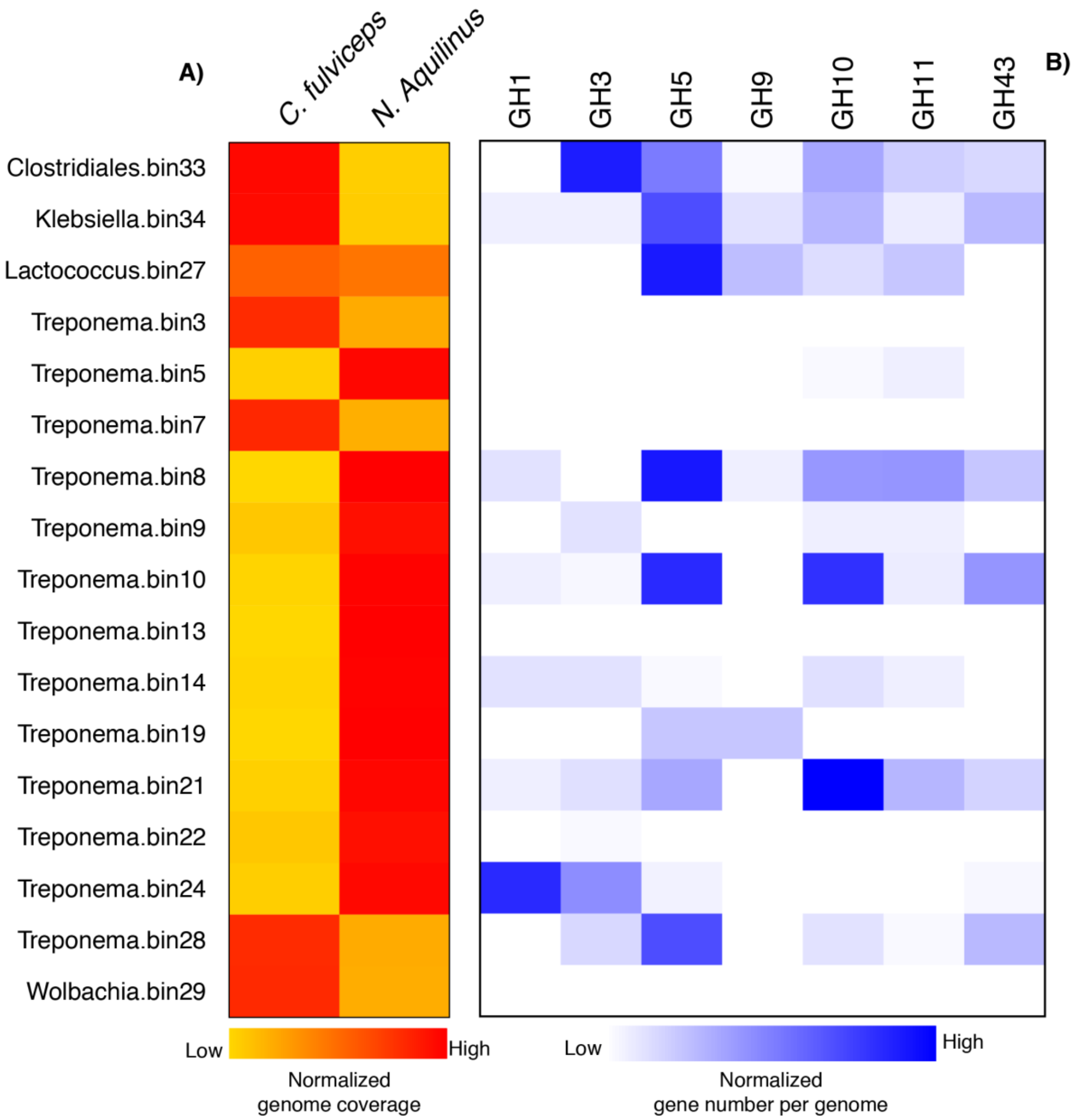

Supplement: Supplementary file 1 — Supplementary material. [file 41598_2020_60850_MOESM1_ESM.pdf]
